# Gut Microbiota Associated With Different Sea Lamprey (*Petromyzon marinus*) Life Stages

**DOI:** 10.3389/fmicb.2021.706683

**Published:** 2021-09-03

**Authors:** Prince P. Mathai, Muruleedhara N. Byappanahalli, Nicholas S. Johnson, Michael J. Sadowsky

**Affiliations:** ^1^BioTechnology Institute, University of Minnesota, St. Paul, MN, United States; ^2^U.S. Geological Survey, Great Lakes Science Center, Lake Michigan Ecological Research Station, Chesterton, IN, United States; ^3^U.S. Geological Survey, Great Lakes Science Center, Hammond Bay Biological Station, Millersburg, MI, United States; ^4^Department of Soil, Water and Climate, University of Minnesota, St. Paul, MN, United States; ^5^Department of Plant and Microbial Biology, University of Minnesota, St. Paul, MN, United States

**Keywords:** sea lamprey, *Petromyzon marinus*, gut microbiota, life stages, microbial community structure

## Abstract

Sea lamprey (SL; *Petromyzon marinus*), one of the oldest living vertebrates, have a complex metamorphic life cycle. Following hatching, SL transition into a microphagous, sediment burrowing larval stage, and after 2–10+ years, the larvae undergo a dramatic metamorphosis, transforming into parasitic juveniles that feed on blood and bodily fluids of fishes; adult lamprey cease feeding, spawn, and die. Since gut microbiota are critical for the overall health of all animals, we examined the microbiota associated with SLs in each life history stage. We show that there were significant differences in the gut bacterial communities associated with the larval, parasitic juvenile, and adult life stages. The transition from larval to the parasitic juvenile stage was marked with a significant shift in bacterial community structure and reduction in alpha diversity. The most abundant SL-associated phyla were Proteobacteria, Fusobacteria, Bacteroidetes, Verrucomicrobia, Actinobacteria, and Firmicutes, with their relative abundances varying among the stages. Moreover, while larval SL were enriched with unclassified Fusobacteriaceae, unclassified Verrucomicrobiales and Cetobacterium, members of the genera with fastidious nutritional requirements, such as *Streptococcus*, *Haemophilus*, *Cutibacterium*, *Veillonella*, and *Massilia*, were three to four orders of magnitude greater in juveniles than in larvae. In contrast, adult SLs were enriched with *Aeromonas*, *Iodobacter*, *Shewanella*, and *Flavobacterium*. Collectively, our findings show that bacterial communities in the SL gut are dramatically different among its life stages. Understanding how these communities change over time within and among SL life stages may shed more light on the role that these gut microbes play in host growth and fitness.

## Introduction

Lampreys (Petromyzontiformes) are one of the oldest living groups of vertebrates (Docker et al., 2015). Along with hagfishes, lampreys number over 40 species and comprise the agnathan (jawless) vertebrates (Potter et al., 2015) with a lineage dating back 500 million years (Janvier, 2007). The lamprey species *Petromyzon marinus*, often generically referred to as the sea lamprey (SL), have a complex metamorphic life cycle. Following hatching, SL transition into a microphagous (filter-feeding-like) larval stage that burrow in soft sediment in or near streams. After several years (ranging 2–17) (Dawson et al., 2015), the larvae undergo a dramatic metamorphosis (Manzon et al., 2015), transform into juveniles that increase markedly in size, reaching 30–110 cm, and parasitize fishes in lakes or oceans, primarily feeding on their blood (Potter et al., 2015). Parasitic lampreys ultimately return to streams where they cease feeding, spawn, and die to complete the life cycle (Johnson et al., 2015).

Sea lamprey are endangered in parts of its native range in Europe (Lucas et al., 2020), but are also an invasive pest in the Laurentian Great Lakes (Marsden and Siefkes, 2019). In the Great Lakes, SL feed on the blood of ecologically and economically valued fishes, and because fishes in the Great Lakes are smaller relative to marine fishes, SL often kill their host (Smith and Tibbles, 1980). A binational consortium involving the United States and Canada, which is managed by the Great Lakes Fishery Commission and its partners, has been tasked to develop strategies for controlling SL and reducing their impact on native Great Lakes fish species (Brant, 2019). Barriers are used to block spawning migrations and selective pesticides are used to control larval SL in streams (Siefkes, 2017). In both their native and non-native ranges, improved and more efficient rearing of SL could benefit research and management, and in the Great Lakes, new selective pesticides could improve control (Hume et al., 2020).

Gut microbiota are critical for the overall health of nearly all animals species studied to date (Kostic et al., 2013). Each species has a relatively distinct and coevolved microbiota contributing to host nutrition and health. Diet has a major influence on the diversity and function of the gut microbiota and relationships between diet and microbes have been studied extensively in recent years (David et al., 2014). However, how the gut microbiota of animals change in response to their multistage life histories (i.e., metamorphosis) is relatively understudied (Kohl et al., 2013), especially in hematophagous animals (Tetlock et al., 2012; Maltz et al., 2014; Michel et al., 2018; Zepeda Mendoza et al., 2018). Given this complex physiological life cycle, the gut microbiota of SLs likely significantly changes in concert with changing dietary needs.

The objective of the current study was to identify and characterize the bacterial communities associated with landlocked SL during different life stages using a 16S rRNA gene-based sequencing approach. We hypothesized that bacterial communities would differ among life stages and become less complex and more specialized in parasitic juvenile SL, because they feed exclusively on blood and bodily fluids. A thorough understanding of bacterial communities associated with each SL life stage may help inform restoration of valued populations, as well as control invasive populations.

## Materials and Methods

### Collection of Lamprey and Digestive Samples

Sea lamprey representing each life stage were obtained from the Lake Huron watershed. Equal numbers of SL from each life stage could not be obtained due to difficulty procuring juvenile and adult life stages. Samples of the surrounding water were not collected nor were samples of the skin of SL hosts, although these could be avenues of future research to understand how external sources of microbiota influence the gut microbiota of SLs.

Larval SL were collected *via* backpack electrofishing (ABP-2 backpack electrofishers, ETS Electrofishing Systems, LLC) (Hansen and Jones, 2008) in Bolton Creek (46°15′52.19″N, 83°15′10.60″W), a Lake Huron tributary located in Ontario, Canada on August 6, 2019. Upon capture SL were transported to Hammond Bay Biological Station (HBBS). Larvae were euthanized with an overdose of buffered tricaine methanesulfonate. The intestine from each larva was removed and cut into 5 mm sections. Sectioned intestine was placed in a 2.0 ml microcentrifuge tube, the tube was filled with 1.7 ml RNA-later, and frozen at −20°C. Scalpel blades were changed between lampreys and larva, and tweezers and cutting boards were sterilized with 10% bleach solution. Intestine was sampled from 53 larvae having a mean length of 114 mm (range: 84–133 mm) and mean weight of 2.3 g (range: 1.0–3.4 g). The sex of the larvae was not determined.

Juvenile SL were removed from lake trout (*Salvelinus namaycush*) or Chinook salmon (*Oncorhynchus tshawytscha*) that were angled from Lake Huron near Cheboygan, Michigan (45°52′21.69″N, 84°14′53.26″W). Juveniles were collected from June 30 to August 12, 2019 and were frozen the day of capture. SL were transported to the lab at the HBBS, thawed, the intestines were removed and dissected into nine 5 mm by 5 mm pieces. Three pieces were placed in a 2.0 ml microcentrifuge tube such that three triplicate samples were collected per individual. Tubes were filled with 1.7 ml RNA-later and frozen at −20°C. Scalpel blades were changed between lampreys, and tweezers and cutting boards were sterilized using a 10% solution of bleach (final hypochlorite concentration of ∼0.52%). Intestines were sampled from 7 juvenile SL (21 samples total; 3 from each fish), having a mean length of 263 mm (range: 185–370 mm), and mean weight of 53 g (range: 16–136 g). The sex of the juveniles was not determined.

Adult SL were trapped from the Black Mallard River, Michigan (45°33′13.94″N, 84°08′11.59″W), a tributary to Lake Huron. Adults were captured between April 24 and May 24, 2019. When a lamprey was captured, fecal samples were collected at U.S. Geological Survey, HBBS by applying pressure to the abdomen. A sterile swab was used to collect fecal material and the swab was placed directly in a microcentrifuge tube containing 1.5 ml of RNA-later (ThermoFisher, Waltham, MA, United States). Samples were stored at −20°C until processed. To minimize risk of DNA contamination, samples were collected by personnel wearing latex gloves. The urogenital region of each SL was washed with deionized water prior to swabbing. Fecal samples were obtained from 35 adult SL having a mean length of 477 mm (range: 320–560 mm) and a mean weight of 208 g (range: 69–340 g). Sixty percent (21 of 35) of the captured SL were females.

### Sample Processing, DNA Extraction, and Amplicon Sequencing

After shipping samples on ice to the University of Minnesota (St. Paul, MN, United States), the dissected tissue samples were transferred into PowerBead tubes containing Solution 1 (Qiagen, Germantown, MD, United States) and were ground for 2 min using a sterile micro pestle. DNA was extracted from the suspension using the DNeasy PowerSoil DNA Isolation Kit (Qiagen) and starting with the bead beating step, according to the manufacturer’s instructions. DNA concentrations were measured using a Qubit 2.0 Fluorometer (Waltham, MA, United States) and all samples were stored at −20 °C until further analyzed.

DNA samples (*n* = 109) were sequenced at the University of Minnesota Genomics Center (UMGC; Minneapolis, MN, United States) by using universal primers: 515f (5′-GTGCCAGCMGCCGCGGTAA-3′) and 806r (5′-GGACTACHVGGGTWTCTAAT-3′) targeting the V4 region of the 16S rRNA gene as described elsewhere (Gohl et al., 2016). Bar-coded sequencing was performed on the MiSeq platform (Illumina, San Diego, CA, United States) using a 2 × 300-bp paired end protocol.

### Bioinformatics and Statistical Analyses

DNA sequences were analyzed by using QIIME v.1.8.0 (Caporaso et al., 2010). Illumina adapters and low quality regions (<Q30) were removed using Trimmomatic v. 3.2 (Bolger et al., 2014). Reads with less than 75% of the total amplicon length were discarded and high-quality reads were joined in pandaseq using the fastqjoin script (Masella et al., 2012; Aronesty, 2013). Chimeras were identified using UCHIME v. 6.1 (Edgar et al., 2011). A naïve Bayesian classifier was used to classify sequences against the RDP training set v. 9 at an 80% bootstrap confidence score (Wang et al., 2007). Open-reference operational taxonomic units (OTUs) were clustered at 3% dissimilarity (97% similarity) using UCLUST and compared against the SILVA v.132 16S rRNA database using PyNast (Caporaso et al., 2009; Edgar, 2010; Quast et al., 2013). The OTU counts were rarefied to 10,000 sequences per sample for statistical analyses. A total of 23 samples, that either failed sequencing or with <10k reads per sample, were removed prior to statistical analyses, resulting in 86 sequenced samples (larvae = 47, juvenile = 15, adult = 24) that were used in further analyses. These fastq files were deposited in the NCBI Sequence Read Archive under BioProject accession number PRJNA727788.

Statistical analyses was performed using QIIME v.1.8.0 and XLSTAT Ecology v 19.6 (Addinsoft, New York, NY, United States). Alpha diversity measures were calculated using the Chao1, Shannon H, observed species, and Simpson E indices. Bray–Curtis dissimilarity matrices were used for principal coordinate analysis (PCoA). These matrices were also used to assess differences in beta diversity by analysis of similarity (ANOSIM) (Mathai et al., 2020). Canonical correspondence analysis (CCA) was done to visualize the relationships between life stages and taxa. OTUs that were present in >90% of samples within each life stage were classified as members of the core microbiota. Taxa that were overrepresented in each life stage were identified using linear discriminant analysis effect size (LEfSe) analysis (Segata et al., 2011). Functional annotation of taxa was performed using the program “functional annotation of prokaryotic taxa” (FAPROTAX) (Louca et al., 2016). A *p*-value < 0.05 was considered to indicate statistical significance for all tests. A statistical test for influence of individual size and condition was not included due to the limited number of samples.

## Results

**FIGURE 1:**
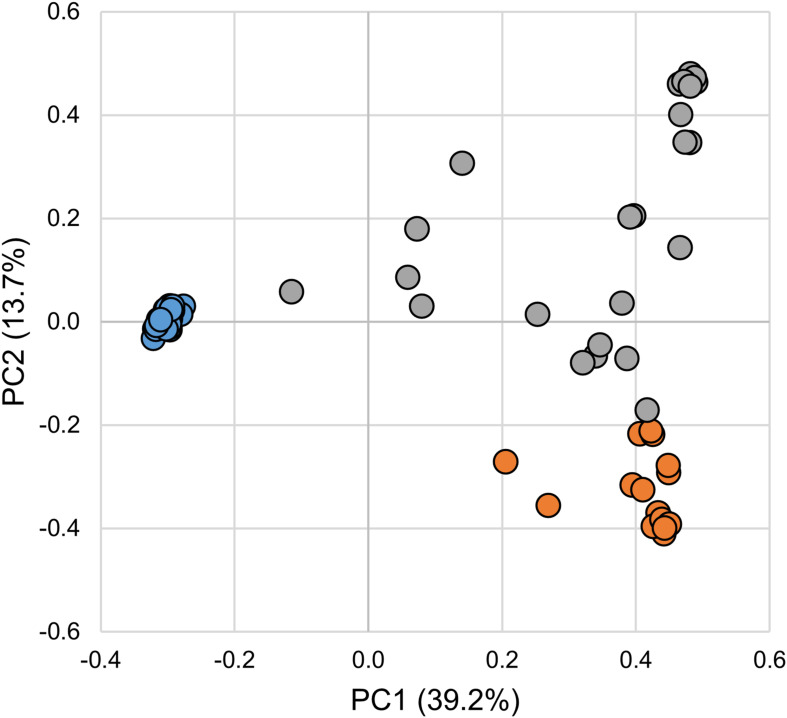
Principal coordinates analysis of microbial communities associated with larval, parasitic, and adult sea lamprey (*Petromyzon marinus*). Each symbol represents one sample. Legend: larval (blue), parasitic (orange), and adult (gray).

**FIGURE 2:**
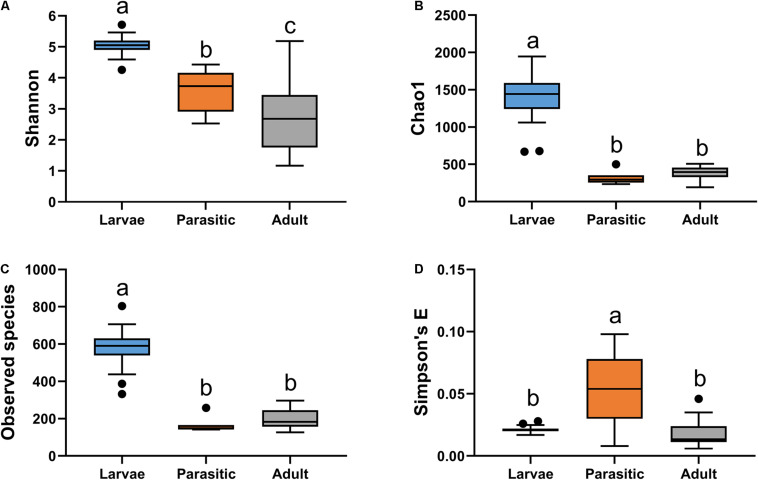
Alpha diversity analysis of microbial communities associated with larval, parasitic, and adult sea lamprey (*Petromyzon marinus*): **(A)** Shannon, **(B)** observed species, **(C)** Chao1, and **(D)** Simpson’s E. Tukey’s HSD post hoc test. On the boxplots, the centerlines show the medians, the bottom and upper limits indicate the 25th and 75th percentiles and the whiskers encompass the 10–90 percentile range; significant differences (*p* < 0.05) illustrated by different letters above bars.

The gut bacterial communities associated with SL larval, parasitic juvenile, and adult life stages differed significantly (ANOSIM *R* = 0.881; *p*-value = 0.001). Notably, the transition from larval to the parasitic juvenile stage was marked with a significant shift in bacterial community structure (Figure 1) and a reduction in alpha diversity indices (Figure 2). Bacterial communities in larvae were significantly more diverse (Shannon) and showed greater species richness (observed OTUs and Chao1), as well as evenness (Simpson’s E) relative to the parasitic and adult SLs.

**FIGURE 3:**
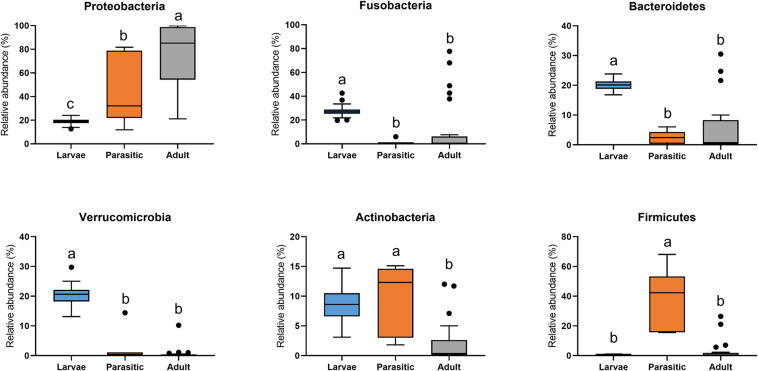
Phylum-level classification of microbial communities associated with larval, parasitic, and adult sea lamprey (*Petromyzon marinus*). All taxa present in ≥5% relative abundance in an individual SL are shown in this graph; significant differences (*p* < 0.05) illustrated by different letters above bars.

**FIGURE 4:**
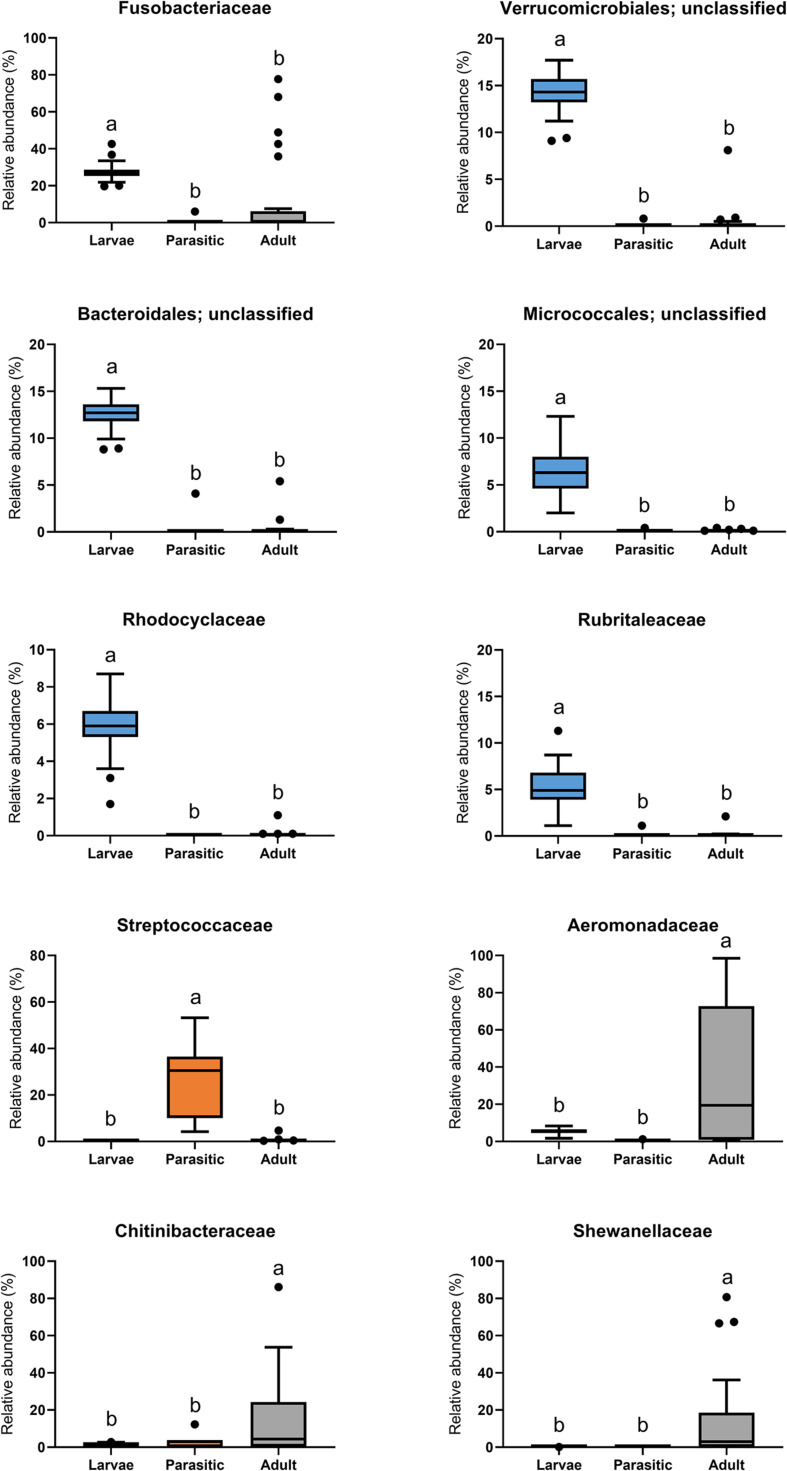
Family-level classification of microbial communities associated with larval, parasitic, and adult sea lamprey (*Petromyzon marinus*). All taxa present in ≥5% relative abundance in an individual SL are shown in this graph.

The most abundant phyla associated with SLs were Proteobacteria, Fusobacteria, Bacteroidetes, Verrucomicrobia, Actinobacteria, and Firmicutes (Figure 3). Members of the phyla Fusobacteria, Firmicutes, and Proteobacteria were overrepresented in larval, parasitic juvenile, and adult stages, respectively. At the family level, larval SLs were enriched for Fusobacteriaceae, unclassified Verrucomicrobiales, unclassified Bacteroidales, unclassified Micrococcales, Rhodocyclaceae, and Rubritaleaceae, whereas Streptococcaceae was enriched in parasitic SLs, and Aeromonadaceae, Chitinibacteraceae, and Shewanellaceae in the adult SLs (Figure 4).

**FIGURE 5:**
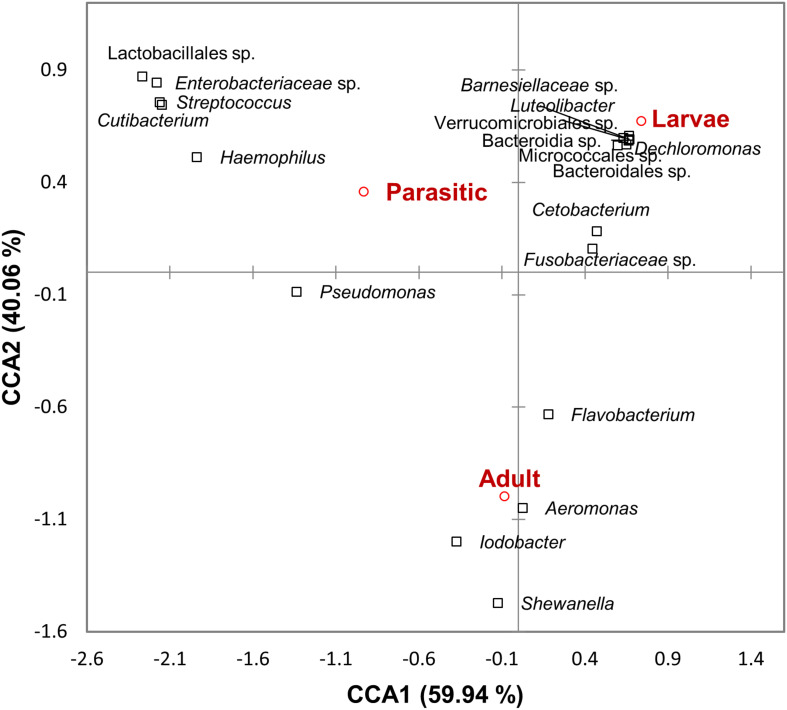
Canonical correspondence analysis ordination diagram correlating microbial taxa and sea lamprey (*Petromyzon marinus*) life stages. The first two axes and the proportion of constraint variance they explain are shown.

Linear discriminant analysis effect size analysis identified significant differences in taxa (at all levels of taxonomic classification) among the larval, parasitic, and adult lamprey stages; only taxa with linear discriminant analysis (LDA) effect sizes ≥3.75 are shown in Table 1. The larval SLs were enriched with members of the unclassified Fusobacteriaceae, unclassified Verrucomicrobiales and *Cetobacterium*. In contrast, members of the genera *Streptococcus*, *Haemophilus*, *Cutibacterium*, *Veillonella*, and *Massilia* were 3–4 orders of magnitude more abundant in the parasitic SLs compared to the larval SLs Other taxa that were dominant, but not significantly enriched during the parasitic stage were: *Pseudomonas*, unclassified Lactobacillales, and unclassified Enterobacteriaceae. In addition, adult SLs were enriched with *Aeromonas*, *Iodobacter*, *Shewanella*, and *Flavobacterium*. Furthermore, CCA confirmed the association of these taxa with each life stage (Figure 5).

**TABLE 1:** Taxa enriched in different life stages of sea lamprey (*Petromyzon marinus*) as determined by LEfSe analysis.

No.	Life stage	LDA score^1^	Relative abundance (%)		
			Larvae	Parasitic	Adult
	**Larvae**				

1	Fusobacteriaceae; unclassified	5.01	23.1 ± 3.3	1.1 ± 3.2	10.3 ± 20.0
2	Verrucomicrobiales; unclassified	4.84	14.6 ± 2.0	0.2 ± 0.6	0.5 ± 1.6
3	*Cetobacterium*	4.30	4.40 ± 0.7	0.2 ± 0.7	1.7 ± 3.1
4	Chitinibacteraceae; unclassified	3.88	1.5 ± 0.5	0.0 ± 0.0	0.0 ± 0.1
5	Rhodocyclaceae; unclassified	3.88	1.5 ± 0.5	0.0 ± 0.0	0.0 ± 0.0
6	Rhodobacteraceae; unclassified	3.80	1.3 ± 0.5	0.1 ± 0.2	1.2 ± 1.9

	**Parasitic juvenile**				

1	*Streptococcus*	5.12	0.0 ± 0.0	28.2 ± 18.5	0.3 ± 1.0
2	*Haemophilus*	4.15	0.0 ± 0.0	2.8 ± 4.6	0.0 ± 0.1
3	*Cutibacterium*	4.11	0.0 ± 0.0	2.6 ± 2.4	0.1 ± 0.1

	**Adult**				

1	*Aeromonas*	5.23	4.3 ± 1.4	0.3 ± 0.9	33.6 ± 35.7
2	*Iodobacter*	4.82	0.0 ± 0.0	3.0 ± 6.7	13.8 ± 21.1
3	*Shewanella*	4.77	0.0 ± 0.0	0.0 ± 0.0	14.6 ± 23.4
4	*Flavobacterium*	4.25	1.3 ± 0.5	0.1 ± 0.2	3.6 ± 7.3
5	Actinobacteria; unclassified	4.22	0.0 ± 0.0	0.0 ± 0.0	0.1 ± 0.5
6	*Rhodococcus*	3.92	0.0 ± 0.0	0.0 ± 0.0	0.2 ± 0.4
7	*Janthinobacterium*	3.85	0.0 ± 0.0	0.0 ± 0.0	0.1 ± 0.2
8	*Brevundimonas*	3.82	0.0 ± 0.0	0.0 ± 0.0	0.5 ± 0.9

*Relative abundance values are relative to the entire microbial community for each life stage. LEfSe, linear discriminant analysis effect size; LDA, linear discriminant analysis. ^1^Analysis was performed at a LDA score ≥3.75.*

A total of 116, 2, and 6 OTUs were identified in ≥90% of the larval, parasitic, and adult SLs, respectively, which were defined as members of their core microbiota (Supplementary Table 1). None of the core OTUs were shared between each life stage.

**FIGURE 6:**
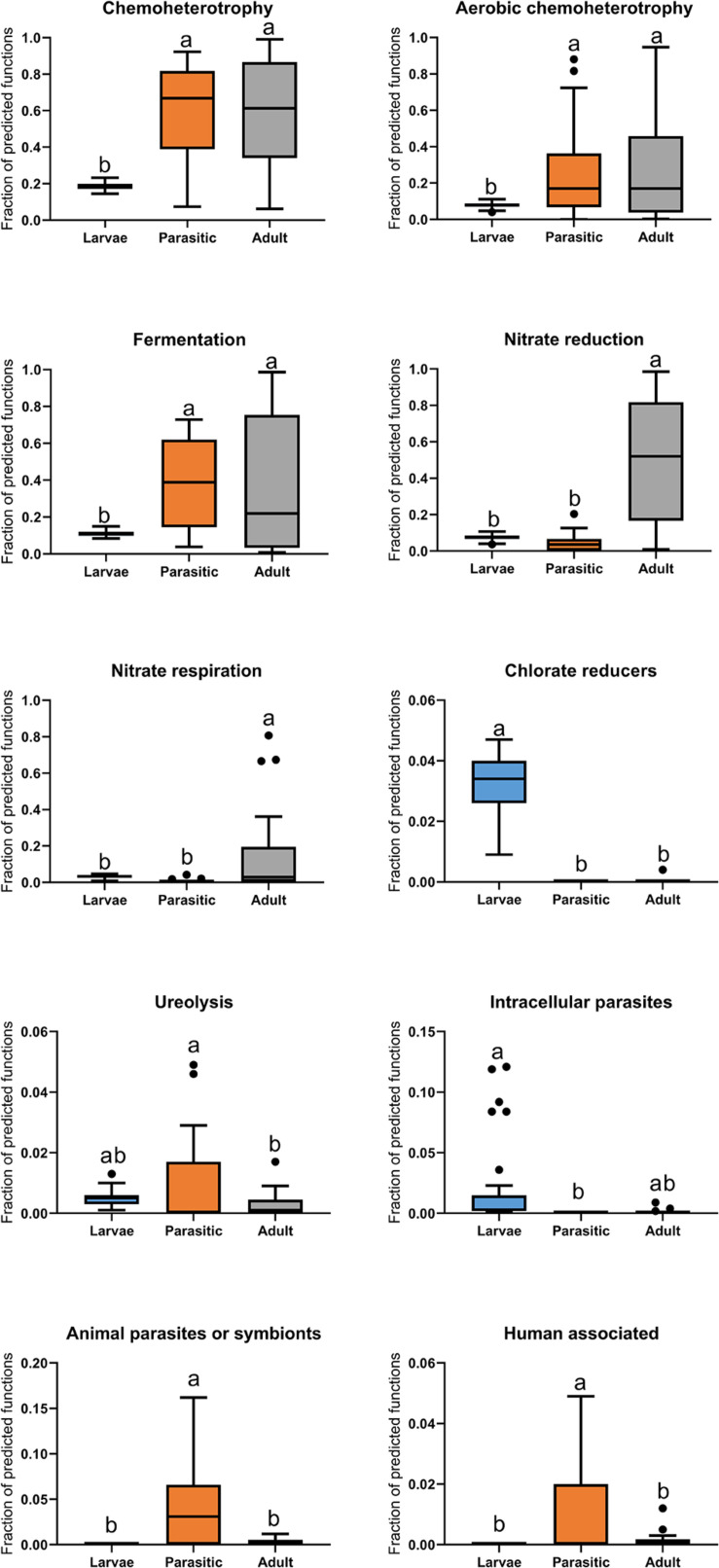
Boxplot of predicted functions based on FAPROTAX database. Tukey’s HSD post hoc test. On the boxplots, the centerlines show the medians, the bottom and upper limits indicate the 25th and 75th percentiles and the whiskers encompass the 10–90 percentile range; significant differences (*p* < 0.05) illustrated by different letters above bars.

Microbial functional groups in larval, parasitic, and adult SLs were predicted by comparing taxonomic information of OTUs against the FAPROTAX database. This analysis revealed that parasitic SLs showed significantly higher relative abundances of animal parasite- and human-associated taxa, as well as those involved in ureolysis, compared to larval and adult SLs (Figure 6). Relative to larvae, both the parasitic and adult SLs had significant increases in taxonomic groups related to heterotrophy and fermentation. Moreover, the adults had a greater proportion of taxa related to nitrate reduction and respiration than the other stages.

## Discussion

Sea lampreys have a complex metamorphic life cycle consisting of three distinct stages each with a unique feeding behavior: (a) larvae typically feed on detritus and algae; (b) juveniles feed on fish blood; and (c) adults essentially cease feeding, spawn, and then die. We hypothesized that distinct intestinal microbiota in SLs likely correspond with their three different life stages. In this study we show that the lamprey gut microbiota change in concert with its dietary needs. For clarity and ease of discussion, the relationship between lamprey life stages and gut bacterial community composition is presented sequentially.

Larval SL are filter feeders burrowing in sediments and slowly draw water across their pharynx, resulting in the trapping and concentration of suspended particles in mucus (Mallatt, 1981). Suspended particles in stream water are diverse and can consist of diatoms, detritus, and bacteria (Sutton and Bowen, 1994; Mundahl et al., 2005). Larvae appear non-selective in capturing particulate matter (Moore and Beamish, 1973), and therefore their diet is diverse and likely varies by season and among inhabited streams. Given the low nutritional value of most of these food sources and potential competition by other aquatic biota, SL’s successful establishment in certain streams may be attributed to their efficient nutrient assimilation efficiencies and digestion (Moore and Beamish, 1973; Mundahl et al., 2005). The diversity of bacterial communities in the larval stage was noteworthy – with taxa representing as many as eight major phyla (e.g., Actinobacteria, Bacteroidetes, Firmicutes, Planctomycetes, Fusobacteria, Proteobacteria) – relative to the parasitic and adult stages (Supplementary Table 1). Moreover, the increased relative abundance of bacterial taxa representing Verrucomicrobiales and Chitinibacteraceae (Table 1), which are capable of degrading complex carbohydrates, suggests that such complex food sources are available to the larvae while growing in streams (Molinari et al., 2007; Sichert et al., 2020).

Juvenile parasitic lamprey are opportunist (Silva et al., 2014; Happel et al., 2017), feeding on fishes ranging from small Catostomidae (150 mm) in the Great Lakes to Blue-(*Prionace glauca*; Moyer et al., 2020) and basking-sharks (*Cetorhinus maximus*; Wilkie et al., 2004) in marine waters. As much as 98% of the parasitic lamprey’s diet is blood, with the remainder being products of tissue cytolysis and other bodily fluids (Farmer, 1980). Conversion efficiency of the blood meal diet is high (nearly 40% at 10°C), with growth rates highest between 15 and 20°C (Farmer, 1980). These drastic changes in the digestive system during metamorphosis, have led to speculation that there is selection and retention of specific microbiota whose functional roles are consistent with lamprey dietary needs (Tetlock et al., 2012). Digestion of blood likely requires a different set of microbial communities than other feeding strategies, given its lack of essential vitamins, high osmolarity, and toxic levels of iron and urea (Song et al., 2019). Predictive functional profiling *via* FAPROTAX database revealed an enrichment of animal parasite- and human-associated bacterial taxa in parasitic SLs compared to the larval and adult SLs (Figure 6). It should be cautioned, however, that the predicted functions are tentative and somewhat speculative pending further isolation and biochemical characterization of individual taxa is achieved.

We found a significant metamorphosis-induced shift in bacterial community structure (Figure 5), as well as enrichment of certain bacterial genera, such as *Streptococcus*, *Haemophilus*, and *Cutibacterium* (Table 1). These are all fastidious bacteria that have specific nutritional needs and are typically grown (*in vitro*) in complex media supplemented with blood products. Bacterial genera, such as *Streptococcus*, and *Hemophilus*, which are capable of hemolytic activity (Fink and Geme, 2006; Toit et al., 2014), are likely to assist in the digestion of blood and bodily fluids in parasitic lamprey. The increased relative abundance of these taxa could also partly explain the reduction of the alpha diversity indices within parasitic SLs compared to larval SLs (Figure 2). Tetlock et al. (2012) reported the isolation of *Aeromonas* species capable of hemolysis from the gut of parasitic SL. However, *Aeromonas* were not detected in juvenile SL. In our study, none of the core OTUs in parasitic stage samples were shared with those from larval stage (Supplementary Table 1), clearly indicating that the transition from larval to the parasitic juvenile stage is accompanied by concurrent selection of bacterial taxa matching their dietary needs.

During the transition from the juvenile to adult stage, the intestine atrophies and the space once occupied by the deteriorated intestine is replaced with gonad (Applegate, 1950) in preparation of spawning. At this point, the SLs stop feeding. This transition was accompanied by significant shift in microbial community structure, as well as the enrichment of certain bacterial genera, including *Aeromonas*, *Iodobacter*, *Shewanella*, and *Flavobacterium* (Table 1). Drastic changes in gut microbiota during a fasting stage has been observed in many animals such as mice, alligators, and pythons (Costello et al., 2010; Keenan et al., 2013; Zarrinpar et al., 2014). While the lack of nutrient availability during the fasting state has been shown to result in the enrichment of bacterial taxa that can degrade host mucin glycans (Derrien et al., 2004), it is also possible that some residual populations are carried over from the parasitic stage or from stream bacteria in the surrounding environment. Frequent samplings across SL life stages will help better define the core bacterial communities during and between the transitions.

## Conclusion

Results of this study show that the gut bacterial communities associated with each SL life stage were significantly different, reflecting their feeding behavior and nutritional requirements. While it is expected that metamorphosis would lead to significant shifts in bacterial community structure, post metamorphic events, such as those present in the transition from parasitic juvenile to spawning adult were equally dramatic. The significantly reduced alpha diversity in gut microbiota observed during parasitic phase appears to be related to the SL’s specialized blood-feeding behavior. The functional composition of the gut microbiota present in the adult is more difficult to reconcile with nutritional and energy requirements needed for reproduction during the non-feeding stage. More work needs to be done to elucidate the functional role of gut microbes during each life stage. This information may reveal new opportunities or targets for developing SL-specific control tools (Thresher et al., 2018), describing differences in growth and survival among lamprey populations (Lucas et al., 2020), or for hatchery rearing protocols for native species requiring restoration (Lampman et al., 2020).

## Data Availability Statement

The datasets presented in this study can be found in online repositories. The names of the repository/repositories and accession number(s) can be found below: https://www.ncbi.nlm.nih.gov/, PRJNA727788.

## Ethics Statement

Sea lamprey were captured and maintained in conditions as recommended under the American Fisheries Society Guidelines for the Use of Fishes in Research (2014).

## Author Contributions

PM, MB, NJ, and MS conceived of the studies, wrote, and edited the manuscript. NJ obtained the samples. MB and PM prepared the samples. PM analyzed the data. All authors contributed to the article and approved the submitted version.

## Conflict of Interest

The authors declare that the research was conducted in the absence of any commercial or financial relationships that could be construed as a potential conflict of interest.

## Publisher’s Note

All claims expressed in this article are solely those of the authors and do not necessarily represent those of their affiliated organizations, or those of the publisher, the editors and the reviewers. Any product that may be evaluated in this article, or claim that may be made by its manufacturer, is not guaranteed or endorsed by the publisher.
