# Corrigendum: Gut Microbiota Associated With Different Sea Lamprey (*Petromyzon marinus*) Life Stages

**DOI:** 10.3389/fmicb.2021.807068

**Published:** 2021-12-13

**Authors:** Prince P. Mathai, Muruleedhara N. Byappanahalli, Nicholas S. Johnson, Michael J. Sadowsky

**Affiliations:** ^1^BioTechnology Institute, University of Minnesota, St. Paul, MN, United States; ^2^U.S. Geological Survey, Great Lakes Science Center, Lake Michigan Ecological Research Station, Chesterton, IN, United States; ^3^U.S. Geological Survey, Great Lakes Science Center, Hammond Bay Biological Station, Millersburg, MI, United States; ^4^Department of Soil, Water and Climate, University of Minnesota, St. Paul, MN, United States; ^5^Department of Plant and Microbial Biology, University of Minnesota, St. Paul, MN, United States

**Keywords:** sea lamprey, *Petromyzon marinus*, gut microbiota, life stages, microbial community structure

In the original article, there was an error. The **ETHICS STATEMENT** was incorrect as follows, the **first sentence should be deleted, and the sentence revised for clarity as shown below**:

Ethical review and approval was not required for the animal study because USGS approved this study. Sea lamprey were captured and maintained in conditions as recommended under the according to their life stage according to the American Fisheries Society Guidelines for the Use of Fishes in Research (2014).

## ETHICS STATEMENT

Sea lamprey were captured and maintained in conditions as recommended under the American Fisheries Society Guidelines for the Use of Fishes in Research (2014).

The authors apologize for this error and state that this does not change the scientific conclusions of the article in any way. The original article has been updated.

## Publisher's Note

All claims expressed in this article are solely those of the authors and do not necessarily represent those of their affiliated organizations, or those of the publisher, the editors and the reviewers. Any product that may be evaluated in this article, or claim that may be made by its manufacturer, is not guaranteed or endorsed by the publisher.

