# Supplementary material for: Gut Microbiota Associated With Different Sea Lamprey (Petromyzon marinus) Life Stages
Source: Front Microbiol. 2021 Sep 3;12:706683. doi: 10.3389/fmicb.2021.706683 (PMC8446677; doi:10.3389/fmicb.2021.706683)
Supplement: Supplementary file 1 [file Data_Sheet_1.docx]

**SUPPLEMENTAL INFORMATION**

**Gut Microbiota Associated with Different Sea Lamprey (*Petromyzon marinus*) Life Stages**

Prince P. Mathai^1^, Muruleedhara N. Byappanahalli^2^, Nicholas S. Johnson^3^, and Michael J. Sadowsky^1,4,*^

^1^ BioTechnology Institute, University of Minnesota, St. Paul, MN 55108. ppmathai@umn.edu

^2^ U.S. Geological Survey, Great Lakes Science Center, Lake Michigan Ecological Research Station, Chesterton, IN 46304. byappan@usgs.gov

^3^ U.S. Geological Survey, Great Lakes Science Center, Hammond Bay Biological Station, 11188 Ray Road, Millersburg, MI 49759. njohnson@usgs.gov

^4^ Department of Soil, Water, and Climate, and Department of Plant and Microbial Biology, University of Minnesota, St. Paul, MN 55108. sadowsky@umn.edu

**Correspondence:** Michael J. Sadowsky: 1479 Gortner Ave., 140 Gortner Labs, BioTechnology Institute, University of Minnesota, St. Paul, MN 55108 USA; Tel.: +1 (612) 624-2706; Fax: +1 (612) 625-5780; Email: sadowsky@umn.edu

**Table S1:** Core OTUs (detected in >90% samples from same life stage)

| **LARVAL** | | | | |
| --- | --- | --- | --- | --- |
| **OTU** |  | **Phylum** | **Family** | **Genus** |
| 1 | CU924911.1.1342 | Actinobacteria | *Microtrichaceae* | IMCC26207 |
| 2 | KY486207.1.1253 | Actinobacteria | *Intrasporangiaceae* | - |
| 3 | HM186141.1.1298 | Actinobacteria | *Intrasporangiaceae* | - |
| 4 | JN093022.1.1222 | Actinobacteria | *Promicromonosporaceae* | *Cellulosimicrobium* |
| 5 | New.ReferenceOTU87 | Actinobacteria | *Rarobacteraceae* | *Rarobacter* |
| 6 | KC554424.1.1514 | Actinobacteria | *Rarobacteraceae* | *Rarobacter* |
| 7 | AB056129.1.1493 | Actinobacteria | *-* | - |
| 8 | New.ReferenceOTU204 | Actinobacteria | *-* | - |
| 9 | AY948355.1.1257 | Actinobacteria | *-* | - |
| 10 | HM186247.1.1335 | Actinobacteria | *Nocardioidaceae* | *Nocardioides* |
| 11 | AY921964.1.1378 | Actinobacteria | *Propionibacteriaceae* | *Propionicicella* |
| 12 | AY928208.1.1318 | Actinobacteria | *Propionibacteriaceae* | *Propionicicella* |
| 13 | CBVB010000006.254456.255954 | Bacteroidetes | *Bacteroidaceae* | *Bacteroides* |
| 14 | GQ360021.1.1486 | Bacteroidetes | *Barnesiellaceae* | uncultured |
| 15 | KC358008.1.1260 | Bacteroidetes | *Barnesiellaceae* | uncultured |
| 16 | New.ReferenceOTU5 | Bacteroidetes | *Barnesiellaceae* | uncultured |
| 17 | New.CleanUp.ReferenceOTU0 | Bacteroidetes | *Barnesiellaceae* | uncultured |
| 18 | EF111176.1.1274 | Bacteroidetes | *Paludibacteraceae* | *Paludibacter* |
| 19 | GQ360012.1.1488 | Bacteroidetes | *-* | - |
| 20 | New.ReferenceOTU2 | Bacteroidetes | *-* | - |
| 21 | New.ReferenceOTU47 | Bacteroidetes | *-* | - |
| 22 | New.ReferenceOTU154 | Bacteroidetes | *-* | - |
| 23 | KX505863.1.1376 | Bacteroidetes | *Flavobacteriaceae* | *Flavobacterium* |
| 24 | KP681249.1.1207 | Bacteroidetes | *Flavobacteriaceae* | *Flavobacterium* |
| 25 | LN870852.1.1382 | Bacteroidetes | *Flavobacteriaceae* | *Flavobacterium* |
| 26 | New.ReferenceOTU42 | Bacteroidetes | *-* | - |
| 27 | New.ReferenceOTU3 | Bacteroidetes | *-* | - |
| 28 | New.ReferenceOTU83 | Bacteroidetes | *-* | - |
| 29 | New.ReferenceOTU28 | Bacteroidetes | *-* | - |
| 30 | New.ReferenceOTU147 | Epsilonbacteraeota | *Helicobacteraceae* | *Helicobacter* |
| 31 | New.ReferenceOTU198 | Firmicutes | *Ruminococcaceae* | - |
| 32 | New.ReferenceOTU94 | Firmicutes | *-* | - |
| 33 | JX262569.1.1478 | Fusobacteria | *Fusobacteriaceae* | *Cetobacterium* |
| 34 | New.ReferenceOTU111 | Fusobacteria | *Fusobacteriaceae* | *Cetobacterium* |
| 35 | New.ReferenceOTU35 | Fusobacteria | *Fusobacteriaceae* | *Cetobacterium* |
| 36 | KC357923.1.1249 | Fusobacteria | *Fusobacteriaceae* | - |
| 37 | New.ReferenceOTU207 | Fusobacteria | *Fusobacteriaceae* | - |
| 38 | New.ReferenceOTU1 | Fusobacteria | *Fusobacteriaceae* | - |
| 39 | New.ReferenceOTU123 | Fusobacteria | *Fusobacteriaceae* | - |
| 40 | New.ReferenceOTU152 | Fusobacteria | *Fusobacteriaceae* | - |
| 41 | New.ReferenceOTU14 | Patescibacteria | *-* | - |
| 42 | New.ReferenceOTU200 | Patescibacteria | *-* | - |
| 43 | KM251014.1.1455 | Planctomycetes | *Pirellulaceae* | Pir4 lineage |
| 44 | FPLS01025198.32.1511 | Planctomycetes | *Pirellulaceae* | uncultured |
| 45 | HQ827902.1.1477 | Planctomycetes | *Pirellulaceae* | uncultured |
| 46 | FPLL01007562.9.1503 | Planctomycetes | *Pirellulaceae* | uncultured |
| 47 | FPLS01018017.9.1494 | Planctomycetes | *Pirellulaceae* | uncultured |
| 48 | JF265791.1.1314 | Proteobacteria | *Reyranellaceae* | *Reyranella* |
| 49 | KJ615121.1.1238 | Proteobacteria | *Beijerinckiaceae* | *Bosea* |
| 50 | KC682980.1.1480 | Proteobacteria | *Rhizobiaceae* | - |
| 51 | CU926988.1.1305 | Proteobacteria | *Rhizobiaceae* | - |
| 52 | HM069053.1.1447 | Proteobacteria | Rhizobiales Incertae Sedis | uncultured |
| 53 | EU937911.1.1425 | Proteobacteria | *Rhodobacteraceae* | *Pseudorhodobacter* |
| 54 | MLJW01003649.603.1987 | Proteobacteria | *Rhodobacteraceae* | *Pseudorhodobacter* |
| 55 | CU919094.1.1285 | Proteobacteria | *Rhodobacteraceae* | *Pseudorhodobacter* |
| 56 | CU920376.2.1285 | Proteobacteria | *Rhodobacteraceae* | - |
| 57 | FPLP01002290.16.1460 | Proteobacteria | *Rhodobacteraceae* | - |
| 58 | FQ659763.1.1283 | Proteobacteria | *Rhodobacteraceae* | - |
| 59 | FQ659285.2.1282 | Proteobacteria | *Rhodobacteraceae* | - |
| 60 | CU920678.1.1283 | Proteobacteria | *Rhodobacteraceae* | - |
| 61 | HG917679.1.1385 | Proteobacteria | *Rhodobacteraceae* | - |
| 62 | JX222044.1.1296 | Proteobacteria | *Desulfobulbaceae* | *Desulforhopalus* |
| 63 | JX224970.1.1209 | Proteobacteria | *Desulfobulbaceae* | - |
| 64 | GQ133047.1.1317 | Proteobacteria | *Desulfomicrobiaceae* | *Desulfomicrobium* |
| 65 | AUBQ01000027.3435.4965 | Proteobacteria | *Desulfovibrionaceae* | *Desulfovibrio* |
| 66 | HM274460.1.1355 | Proteobacteria | *Oligoflexaceae* | *Oligoflexus* |
| 67 | FJ494891.1.1206 | Proteobacteria | *Aeromonadaceae* | *Aeromonas* |
| 68 | KJ806427.1.1205 | Proteobacteria | *Aeromonadaceae* | *Aeromonas* |
| 69 | KP975269.1.1288 | Proteobacteria | *Aeromonadaceae* | *Aeromonas* |
| 70 | HM779393.1.1490 | Proteobacteria | *Aeromonadaceae* | *Aeromonas* |
| 71 | New.ReferenceOTU21 | Proteobacteria | *Aeromonadaceae* | *Aeromonas* |
| 72 | HQ860721.1.1421 | Proteobacteria | *Aeromonadaceae* | *Tolumonas* |
| 73 | EF679186.1.1503 | Proteobacteria | *Aeromonadaceae* | - |
| 74 | GU356341.1.1408 | Proteobacteria | *Aeromonadaceae* | - |
| 75 | KJ808055.1.1503 | Proteobacteria | *Burkholderiaceae* | - |
| 76 | KF841301.1.1499 | Proteobacteria | *Burkholderiaceae* | - |
| 77 | New.ReferenceOTU6 | Proteobacteria | *Chitinibacteraceae* | - |
| 78 | CU926684.1.1359 | Proteobacteria | *Rhodocyclaceae* | *Dechloromonas* |
| 79 | DQ676400.1.1436 | Proteobacteria | *Rhodocyclaceae* | *Dechloromonas* |
| 80 | GQ340232.1.1396 | Proteobacteria | *Rhodocyclaceae* | *Dechloromonas* |
| 81 | JN033124.1.1458 | Proteobacteria | *Rhodocyclaceae* | *Propionivibrio* |
| 82 | EF589963.1.1392 | Proteobacteria | *Rhodocyclaceae* | *Propionivibrio* |
| 83 | CU926836.2.1336 | Proteobacteria | *Rhodocyclaceae* | *Propionivibrio* |
| 84 | JF497831.1.1490 | Proteobacteria | *Rhodocyclaceae* | - |
| 85 | FPLP01011700.8.1527 | Proteobacteria | *Rhodocyclaceae* | - |
| 86 | FJ592534.1.1396 | Proteobacteria | *Rhodocyclaceae* | - |
| 87 | DQ640700.1.1418 | Proteobacteria | *Rhodocyclaceae* | - |
| 88 | New.ReferenceOTU201 | Proteobacteria | *Rhodocyclaceae* | - |
| 89 | New.ReferenceOTU209 | Proteobacteria | *Rhodocyclaceae* | - |
| 90 | New.ReferenceOTU13 | Proteobacteria | *-* | - |
| 91 | New.ReferenceOTU58 | Proteobacteria | *-* | - |
| 92 | LT745987.1.1567 | Proteobacteria | *Enterobacteriaceae* | - |
| 93 | KY476123.1.1200 | Proteobacteria | *Pseudomonadaceae* | *Pseudomonas* |
| 94 | EF111213.1.1286 | Proteobacteria | *Xanthomonadaceae* | *Arenimonas* |
| 95 | KC633561.1.1359 | Proteobacteria | *Xanthomonadaceae* | *Arenimonas* |
| 96 | GQ379552.1.1235 | Proteobacteria | *Xanthomonadaceae* | *Thermomonas* |
| 97 | New.ReferenceOTU68 | Tenericutes | *Mycoplasmataceae* | - |
| 98 | New.ReferenceOTU8 | Verrucomicrobia | *-* | - |
| 99 | New.ReferenceOTU120 | Verrucomicrobia | *-* | - |
| 100 | KP101281.1.1443 | Verrucomicrobia | *Rubritaleaceae* | *Luteolibacter* |
| 101 | CABV01003337.48680.50227 | Verrucomicrobia | *Rubritaleaceae* | *Luteolibacter* |
| 102 | FJ264561.1.1501 | Verrucomicrobia | *Rubritaleaceae* | *Luteolibacter* |
| 103 | AB630914.1.1476 | Verrucomicrobia | *Rubritaleaceae* | *Luteolibacter* |
| 104 | GQ406199.1.1513 | Verrucomicrobia | *Rubritaleaceae* | *Luteolibacter* |
| 105 | JQ319003.1.1407 | Verrucomicrobia | *Rubritaleaceae* | *Luteolibacter* |
| 106 | FJ437989.1.1502 | Verrucomicrobia | *Rubritaleaceae* | *Luteolibacter* |
| 107 | New.ReferenceOTU79 | Verrucomicrobia | *Rubritaleaceae* | *Luteolibacter* |
| 108 | New.ReferenceOTU92 | Verrucomicrobia | *Rubritaleaceae* | *Luteolibacter* |
| 109 | GQ396806.1.1512 | Verrucomicrobia | *Verrucomicrobiaceae* | uncultured |
| 110 | New.ReferenceOTU0 | Verrucomicrobia | *-* | - |
| 111 | New.ReferenceOTU179 | Verrucomicrobia | *-* | - |
| 112 | New.ReferenceOTU75 | - | *-* | - |
| 113 | New.ReferenceOTU61 | - | - | - |
| 114 | New.ReferenceOTU11 | - | - | - |
| 115 | New.ReferenceOTU26 | - | - | - |
| 116 | New.ReferenceOTU214 | - | - | - |
| **PARASITIC** | | | | |
| **OTU** |  | **Phylum** | **Family** | **Genus** |
| 1 | KY003111.1.1207 | Firmicutes | *Streptococcaceae* | *Streptococcus* |
| 2 | KF063662.1.1249 | Actinobacteria | *Propionibacteriaceae* | *Cutibacterium* |
| **ADULT** | | | | |
| **OTU** |  | **Phylum** | **Family** | **Genus** |
| 1 | JQ450757.1.1319 | Proteobacteria | *Rhodobacteraceae* | - |
| 2 | CU926199.1.1283 | Proteobacteria | *Rhodobacteraceae* | - |
| 3 | FJ494891.1.1206 | Proteobacteria | *Aeromonadaceae* | *Aeromonas* |
| 4 | KJ806427.1.1205 | Proteobacteria | *Aeromonadaceae* | *Aeromonas* |
| 5 | KY476123.1.1200 | Proteobacteria | *Pseudomonadaceae* | *Pseudomonas* |
| 6 | EU705610.1.1201 | Proteobacteria | *Caulobacteraceae* | *Brevundimonas* |
